# Targeted therapies in the management of advanced medullary thyroid cancer

**DOI:** 10.1093/oncolo/oyag300

**Published:** 2026-07-29

**Authors:** Hameeda Arif Arain, Azeem Izhar, Rachael Caretti, Herbert Chen, Danica M Vodopivec

**Affiliations:** Division of Breast and Endocrine Surgery, Department of Surgery, University of Alabama at Birmingham, Birmingham, AL 35233, United States; Division of Breast and Endocrine Surgery, Department of Surgery, University of Alabama at Birmingham, Birmingham, AL 35233, United States; Division of Breast and Endocrine Surgery, Department of Surgery, University of Alabama at Birmingham, Birmingham, AL 35233, United States; Division of Breast and Endocrine Surgery, Department of Surgery, University of Alabama at Birmingham, Birmingham, AL 35233, United States; Division of Endocrinology, Diabetes and Metabolism, University of Alabama at Birmingham, Birmingham, AL 35233, United States

**Keywords:** thyroid cancer, medullary, vandetanib, selpercatinib, pralsetinib, cabozantinib, mutation

## Abstract

**Background:**

Medullary thyroid carcinoma (MTC) is a rare neuroendocrine tumor that contributes disproportionately to thyroid cancer–related mortality. Historically, systemic treatment options for advanced MTC were limited due to poor responsiveness to radioactive iodine and conventional chemotherapy. The development of targeted therapies has significantly transformed disease management.

**Methods:**

This narrative review summarizes current evidence on targeted therapies for advanced MTC, including multikinase inhibitors (MKIs), selective RET inhibitors, and emerging treatment strategies. Literature on efficacy, safety, resistance mechanisms, and novel therapeutic approaches was evaluated.

**Results:**

First-generation MKIs, including vandetanib and cabozantinib, demonstrated clinical benefit by targeting RET and angiogenic pathways, though off-target toxicities limited tolerability. More recently, selective RET inhibitors, such as selpercatinib and pralsetinib, have shown high response rates and improved safety profiles, establishing them as preferred first-line therapies for RET-mutant MTC. However, pralsetinib’s recent market withdrawal due to regulatory challenges highlights the need for additional options. Emerging therapies, including BOS172738, SY-5007, and peptide receptor radionuclide therapy, show promising early results. Resistance mechanisms, including gatekeeper and solvent front mutations, remain a challenge. Advances in clinical biomarkers and novel approaches, such as CAR-T cell therapy, may further support personalized treatment.

**Conclusion:**

Targeted therapies have reshaped the management of advanced MTC, with selective RET inhibitors offering improved efficacy and tolerability. Continued research is essential to overcome resistance, expand therapeutic options, and improve patient outcomes.

Implications for Practice This review highlights the shift from broad multikinase inhibitors to selective RET inhibitors in the management of MTC, offering improved efficacy and tolerability. Clinicians should consider selective RET inhibitors as first-line therapy for *RET*-mutant MTC, monitor for resistance mutations, and remain informed on emerging agents to optimize long-term outcomes.

## Introduction

Medullary thyroid carcinoma (MTC) is a neuroendocrine malignancy that arises from the thyroid parafollicular C-cells and represents 3-5% of all thyroid cancers.^1^^,^^2^ While MTC accounts for only a minority of all thyroid cancer diagnoses, it causes a disproportionate 13% of all deaths from thyroid cancer.^3^ Approximately 25% of MTCs are hereditary (MEN2A/2B syndromes with germline *RET* mutations), whereas 75% are sporadic, often driven by activating somatic *RET* (about 45% of somatic cases), and occasionally RAS mutations (but with less frequency).^4^ Parafollicular C-cells secrete multiple biogenic amines with calcitonin (Ctn) and carcinoembryonic antigen (CEA) is used as tumor markers in MTC.^5^ The clinical behavior of MTC is variable: localized disease is often indolent, but advanced (metastatic) MTC has a poor prognosis (5-year survival ≈43%).^6^

MTC does not uptake radioactive iodine, and conventional chemotherapy has limited activity.^4^ Thus, systemic treatment options for advanced progressive MTC were historically inadequate, and new approaches were needed. The identification of *RET* and other oncogenic tyrosine kinase pathways led to the use of targeted therapies in MTC.^7^ Multikinase tyrosine kinase inhibitors (MKIs) that block RET along with angiogenic receptors were the first systemic agents to show benefit. Vandetanib (inhibiting RET, VEGFR, EGFR) and cabozantinib (inhibiting RET, VEGFR2, MET) significantly prolonged progression-free survival (PFS) in phase III trials of advanced MTC, leading to their FDA approval for locally unresectable, or progressive advanced metastatic disease.^8^ These MKIs provided proof that targeting *RET* pathways can alter disease course, although their broad kinase inhibition causes substantial off-target toxicity.^9^^,^^10^

Selective RET inhibitors (pralsetinib and selpercatinib) have transformed the treatment paradigm for *RET*-mutant MTC. These highly potent RET-specific tyrosine kinase inhibitors (TKIs) achieved high response rates with reduced off-target effects.^11^ These agents have demonstrated durable remissions in both TKI-naïve and TKI-pretreated patients.^8^ Selective RET inhibitors thus now represent standard first-line therapy for *RET*-driven advanced MTC.^12^ However, pralsetinib indication in MTC was voluntary withdrawn by the drug company due to inability to fulfill the confirmatory phase III study.^13^

The therapeutic landscape for advanced MTC is rapidly evolving, with new drugs and trials continually emerging. This narrative review aims to evaluate the spectrum of targeted therapies for advanced MTC, focusing on the clinical efficacy and safety of multikinase inhibitors, selective RET inhibitors, and emerging therapeutic options. Furthermore, it seeks to highlight existing gaps in the literature and propose areas for future research to optimize therapeutic outcomes and improve the quality of life for patients with this challenging malignancy.

## Methods

A comprehensive literature search of PubMed, Embase, and the Cochrane Library was conducted using keywords related to (medullary thyroid carcinoma) AND (advanced) AND (targeted therapy). Articles published in English between January 2005 and June 2025 were considered.

The first and second authors independently screened titles and abstracts for eligibility. Full texts of potentially relevant studies were reviewed in detail, and any disagreements were resolved through discussion. Case reports, animal studies, and studies lacking specific outcomes related to MTC were excluded.

### Current targeted therapies approved for use in MTC

#### Multikinase inhibitors

Vandetanib and cabozantinib were the first MKIs approved for advanced MTC, targeting RET along with VEGFR and other kinases.^14^ In placebo-controlled phase III trials, these agents significantly improved PFS and objective response rates (ORR) compared to no treatment. Table 1 summarizes key efficacy outcomes.

**Table 1.** Key clinical trials of targeted therapies in advanced medullary thyroid carcinoma.

Trial (drug)	Phase/design	Patients (advanced MTC)	Key efficacy outcomes (median PFS, ORR)
**ZETA (Vandetanib)** ^16^	Phase III, randomized vs placebo	*n* = 331; progressive locally advanced or metastatic MTC (no prior systemic therapy). Cross-over from placebo to the active treatment in progressing patients.	PFS 30.5 vs 19.3 (HR, 0.46; 95% CI, 0.31-0.69; *P* < .001) ORR 45% (vs 13% placebo)
**EXAM (Cabozantinib)** ^21^	Phase III, randomized vs placebo	*n* = 330; progressive metastatic MTC (≤1 prior TKI allowed). Required radiological progression before enrollment.	PFS 11.2 vs 4.0 (HR, 0.28; 95% CI, 0.19-0.40; *P* < .001) ORR 28% (vs 0% placebo).
**LIBRETTO-001 (Selpercatinib)** ^24^	Phase I/II, single-arm	*n* = 837 (411 had *RET*-mutant MTC) Eligible patients had advanced disease that have progressed following prior treatment or had no acceptable alternative treatment options. Radiographic tumor progression was not a definite inclusion criterion.	ORR 77.6% (previously treated) ORR 82.5% (treatment naïve)
**LIBRETTO-531 (Selpercatinib)** ^25^	Phase III, randomized vs cabozantinib or vandetanib	*n* = 291; progressive advanced RET-mutant MTC; treatment-naïve to kinase inhibitors	Median PFS not reached vs 16.8 months (HR 0.28, 95% CI 0.16-0.48; *P* < .001) Overall response: 69% vs 39% (selpercatinib vs control)
**ARROW (Pralsetinib)** ^26^	Phase I/II, single arm	*n* = 142 (122 with RET-mutant MTC; of these, only 76 were response-evaluable patients with baseline measurable disease) Patients with both kinase inhibitor-naive and kinase inhibitor-refractory disease, as well as any number of prior therapies, were eligible. Patients were required to have disease progression within 14 months before enrollment. All subjects required measurable disease at the time of enrollment and a confirmed pathogenic *RET* mutation.	ORR 60% (previously treated) ORR 71% (treatment naïve)
**ALTER 01031 (Anlotinib)** ^29^	Phase II, randomized vs placebo	*n* = 91	PFS 20.7 months vs 11.1 months (HR 0.53, *P* = .029) ORR 48.4%
**Everolimus ± Pasireotide** ^27^	Phase II, 3-arm randomized	*n* = 42	Everolimus (Arm A) PFS1^a^: 8.3 months (95% CI, 3.7-26.3) PFS2^a^: 26.3 months (95% CI, 8.3-NA)
			Pasireotide LAR (Arm B) PFS1: 1.8 months (95% CI, 1.7-15.6) PFS2: 17.5 months (95% CI, 2.1-30.7)
			Combination (Arm C) PFS1: 8.1 months (95% CI, 3.7-13.8) PFS2: 8.1 months (95% CI, 3.7-13.8)

Abbreviations: EXAM, efficacy of XL184 in advanced medullary thyroid cancer; HR, hazard ratio; MTC, medullary thyroid cancer; ORR, objective response rate; PFS, progression-free survival; RET, rearranged during transfection; TKI, tyrosine kinase inhibitor; ZETA, zactima efficacy in thyroid cancer assessment.

a PFS1 (progression-free survival 1): Time from the initiation of the initial treatment (eg, single-agent therapy) to the first documented disease progression or death from any cause; PFS2 (progression-free survival 2): Time from the initiation of the initial treatment to the second documented disease progression or death, specifically for patients who transitioned to combination therapy after progression on initial single-agent therapy.

The Zactima Efficacy in Thyroid Cancer Assessment (ZETA) trial, which evaluated vandetanib in advanced MTC, demonstrated a significant improvement in PFS compared with placebo. Patients receiving vandetanib had a median PFS of around 30 months, in contrast to 19 months for the placebo group (HR 0.46; *P* < .001). Vandetanib also achieved a higher ORR (45% vs 13%) and disease control rate (DCR) (87% vs 71%) compared with placebo, confirming a significant efficacy advantage in this patient population. However, vandetanib did not show a statistically significant improvement in overall survival (OS) compared with the placebo group as patients in the placebo arm were allowed to switch to vandetanib after disease progression, under an open-label design (HR, 1.08; 95% CI, 0.72-1.61; *P* = .71).^8^^,^^15–17^

Kim et al. confirmed vandetanib’s efficacy in routine clinical practice with outcomes comparable to those observed in the ZETA trial.^16^^,^^18^ The study demonstrated an ORR of 42% and a DCR of 83% for advanced MTC, with a median PFS of 25.9 months.

The Efficacy of XL184 in Advanced Medullary Thyorid Cancer (EXAM) trial reported that cabozantinib significantly extended PFS in patients with metastatic MTC. Median PFS was 11.2 months in the treatment arm, longer than the 4.0 months observed in those on placebo (HR ∼0.28; *P* < .001). The ORR with cabozantinib was approximately 28% vs 0% with placebo. Cabozantinib also did not achieve a statistically significant increase in median OS compared to placebo (26.6 months vs 21.1 months, HR 0.85; 95% CI, 0.64-1.12; *P* = .24). However, in a specific subgroup of patients with the RET M918T mutation, cabozantinib demonstrated a significant OS benefit. These patients had a median OS of 44.3 months with cabozantinib compared with 18.9 months in the placebo group (HR 0.60; 95% CI, 0.38-0.94; *P* = .03).

Masaki et al. showed that vandetanib was the predominant first-line MKI for advanced MTC in Japan, used in 62.5% of patients.^19^ This aligns with Japanese guidelines that recommend vandetanib for recurrent or advanced MTC, while cabozantinib was not included due to its lack of approval during the study period. At the time of analysis, selective RET inhibitors like selpercatinib and pralsetinib were not yet approved, highlighting the reliance on MKIs as primary treatment options.

Despite demonstrating significant improvements in PFS, both vandetanib and cabozantinib are associated with notable toxicities. Common adverse events (AEs) reported with vandetanib include diarrhea (57%), rash (53%), dermatitis acneiform/acne (35%), nausea (33%), hypertension (33%), headache (26%), fatigue (24%), decreased appetite (21%), and QT prolongation (14%). Five patients on vandetanib had rare but serious events such as aspiration pneumonia, respiratory arrest, respiratory failure, staphylococcal sepsis, and arrhythmia with cardiac failure. Cabozantinib is similarly associated with gastrointestinal toxicities, hypertension, and fatigue, and carries a black box warning for serious AEs, such as gastrointestinal perforations, hemorrhage, and fistula formation, particularly in patients with prior radiation or tumor invasion into vital structures. These toxicities often necessitate dose reductions, interruptions, or treatment discontinuation, which can ultimately limit therapeutic efficacy.^20^

#### RET kinase inhibitors

Highly selective RET kinase inhibitors have expanded the therapeutic options for advanced MTC. In contrast to vandetanib and cabozantinib, which also inhibit VEGFR and other kinases, selpercatinib and pralsetinib spare non-RET targets. They were designed to overcome *RET* V804 gatekeeper mutations. Results from the trials are summarized below.

In the LIBRETTO-001 phase I/II study, selpercatinib was found to be highly effective, showing ORRs of 77.6% in patients previously treated with TKIs and 82.5% among those who had not received prior systemic therapy for RET-mutant MTC. Median PFS for the previously treated cohort was 41.4 months. RET M918T mutation was the most common *RET* activating mutation. Responses were observed in 3 of the 8 MTC patients with the acquired gatekeeper resistance mutation RET V804.^21^ Treatment-related AEs (TRAEs) with selpercatinib were mostly grade 1–2 and included dry mouth (39%), fatigue (26%), peripheral edema (18%), diarrhea (17%), constipation (16%), nausea (15%), and headache (13%). Grade 3-4 events were less common but included hypertension (12%), increased AST (28%), increased ALT (26%), and prolonged QTc (13%). TRAEs led to dose reduction in 30% and discontinuation in only 2% of patients.^20^

The phase III LIBRETTO-531 trial (Hadoux et al.^22^) compared selpercatinib with cabozantinib or vandetanib in treatment-naïve patients with advanced RET-mutant MTC. Selpercatinib had superior outcomes, with an HR for progression or death of 0.28 (95% CI 0.16-0.48; *P* < .001), median PFS not reached vs 16.8 months with the control group, and higher overall response (69% vs 39%). Treatment discontinuations due to AEs were lower with selpercatinib (4.7% vs 26.8%), highlighting its favorable safety profile.

Findings from the ARROW trial revealed that pralsetinib induced responses in both naïve and previously treated with MKIs (cabozantinib and/or vandetanib) RET-mutant MTC patients. The ORR was 71% among treatment naïve patients (*n* = 15/21; 95% CI 48-89) with 5% CR (*n* = 1/21) and 60% (*n* = 33/55; 95% CI 46-73) with 2% CR (*n* = 1/55) for those with prior exposure to MKIs.^23^ The most common AEs with pralsetinib were musculoskeletal pain (18%), constipation (28%), diarrhea (16%), headache (13%), and dry mouth (12%). Grade ≥3 toxicities included hematologic abnormalities (neutropenia [34%], lymphopenia [34%], anemia [29%]) and hypertension (33%). Pneumonitis was the most frequent serious TRAE (4%). Dose reduction occurred in 46%, interruptions in 54%, and discontinuation in 4% of patients.^20^

Both selpercatinib and pralsetinib seem equally effective for the *RET*-mutant MTC patient population. The distinguishable features are (1) the difference in dosing interval where selpercatinib is given twice a day vs pralsetinib once a day, (2) selpercatinib can be dissolved per specified instructions, and (3) the drug company withdrew pralsetinib for the treatment of *RET*-mutant MTC in June 2023. After the ARROW trial results, the FDA granted pralsetinib an accelerated approval pathway in December 2020 for the treatment of *RET*-mutant MTC. However, full approval required demonstration of clinical benefit in the confirmatory Phase 3 randomized AcceleRET MTC study. Unfortunately, this later study could not be activated to fulfill the post marketing requirement. Pralsetinib can be given to patients, but it is considered as off-label by insurances.

### Ongoing research for potential new targeted therapies for MTC

#### mTOR inhibitor everolimus

Bauman et al.^24^ conducted a trial of everolimus (an mTOR inhibitor) and pasireotide-LAR in patients with advanced thyroid cancers (76% differentiated, 24% medullary). The study included 3 treatment strategies: single-agent everolimus, single-agent pasireotide LAR, and upfront combination therapy with both agents. Sequential treatment strategies were evaluated, with patients transitioning to combination therapy upon progression. No patients achieved an objective response based on RECIST criteria. Median PFS1 was 8.3 months with everolimus alone, comparable to 8.1 months with upfront combination therapy, while pasireotide monotherapy demonstrated limited activity (PFS1 1.8 months). However, sequential therapy resulted in improved outcomes, with a median PFS2 of 26.3 months in patients who received everolimus followed by addition of pasireotide upon progression, compared to 8.1 months in those receiving upfront combination therapy. These findings suggest that sequential or combinatorial endocrine-targeted strategies may enhance the modest efficacy of mTOR inhibition in advanced thyroid cancer.

Lim et al. conducted a multicenter, phase II trial evaluating everolimus in locally advanced or metastatic thyroid cancer of all histologic subtypes, including 9 patients with MTC. Forty patients received oral everolimus 10 mg daily until progression or severe AEs. The primary endpoint was DCR (defined as partial response [PR] + stable disease [SD] ≥ 12 weeks). Among 38 evaluable patients, 2 (5%) achieved PR and 29 (76%) achieved SD, yielding a DCR of 81%. 45% experienced durable SD (≥24 weeks), and median PFS across all histologic subtypes was 47 weeks (95% CI 14.9-78.5). For MTC specifically, no objective radiographic responses were observed, several patients demonstrated ≥50% reductions in Ctn and CEA levels, suggesting potential biochemical activity. In the context of MTC, no objective radiographic responses were seen; however, several patients maintained disease stability, and some demonstrated meaningful biochemical marker declines.^25^ Together with the Bauman et al. combination trial, these findings show that mTOR inhibition, either as monotherapy or in combination, remains an avenue of ongoing research in MTC, particularly in strategies designed to overcome resistance mechanisms.

#### MKIs

Several studies have examined MKIs beyond the established vandetanib/cabozantinib in MTC. Li et al.^26^ reported a randomized, placebo-controlled phase IIB trial (ALTER-01031) of anlotinib (a multitarget TKI against VEGFR/FGFR/PDGFR/c-KIT) in 91 patients with advanced MTC in China. Anlotinib significantly improved median PFS of 20.7 months (vs 11.1 months on placebo) and an ORR of 48.4% on anlotinib (vs 0% on placebo).

Zhao et al.^27^ performed a subgroup analysis of ALTER-01031, focusing on poor prognosis patients (age ≥50 or bone metastases). Anlotinib dramatically improved outcomes in these subgroups: in patients age ≥50, median PFS was 17.5 months (vs 6.8 months on placebo). In patients with bone metastases, median PFS prolonged to 20.7 months and decreased the risk of disease progression by 56%.

In a multicenter phase II trial, Schlumberger et al. evaluated lenvatinib in 59 patients with advanced MTC, with participants included irrespective of whether they received prior VEGFR-directed therapy. The study reported an ORR of 36% (all PRs) and a disease control rate of 80%. Median PFS was 9 months. The safety profile was consistent with VEGFR blockade, with diarrhea (14%), hypertension (7%), and decreased appetite (7%), among the most common toxicities.^28^

Lam et al. investigated sorafenib in a phase II study of 21 patients, including both sporadic and hereditary MTC. Most patients demonstrated SD (several for more than 15 months), and one patient with sporadic disease achieved a PR (6%). The median PFS was 17.9 months. Although the ORR was modest, serum Ctn and CEA levels decreased in the majority of patients, indicating biological activity. Toxicities included diarrhea, rash, hand–foot skin reaction, and hypertension, with one treatment-related death reported, emphasizing the need for careful monitoring.^29^

Bible et al. conducted a multicenter phase II trial of pazopanib in 35 patients with progressive metastatic MTC. PR was seen in 14% of patients with median PFS of 9.4 months and an OS of 19.9 months. The most common AEs were hypertension, fatigue, and diarrhea; 8.6% of patients discontinued therapy due to toxicity, and one treatment-related death was reported.^30^

These MKIs demonstrate variable levels of activity in MTC, with anlotinib and lenvatinib showing encouraging response rates, while sorafenib and pazopanib appear to provide more modest benefit primarily through disease stabilization.

#### RET inhibitors

Recent efforts to overcome resistance to RET inhibitors have led to the development of EP0031 (A400/KL590586), a next-generation RET inhibitor. In a multicenter Phase I study including patients with advanced *RET*-altered tumors, 12 participants had MTC, of whom 7 had previously received RET inhibitors. Among pretreated MTC patients, EP0031 achieved PR in 2 cases with a duration of response (DOR) of 6.6-9.2 months, and SD in 2 cases. RET inhibitor-naïve MTC patients demonstrated objective responses, including 5 PR. The trial also confirmed activity against on-target RET resistance mutations, such as G810R, L730V, and L730I, with reductions and even clearance of circulating tumor DNA. Responses were durable and observed in patients with brain metastases, an area of unmet need. Ongoing Phase II trials across the United States, Europe, UAE, and China will help to define its role relative to established RET inhibitors.^31^

#### BOS172738

BOS172738 is a novel, selective RET inhibitor with nanomolar potency and approximately 300-fold selectivity over VEGFR2, designed to minimize off-target toxicities commonly seen with MKI’s. In a phase 1, open-label, dose-escalation trial (NCT03780517), BOS172738 is being evaluated for safety, tolerability, and preliminary efficacy in patients with advanced solid tumors harboring *RET* alterations. The study includes expansion cohorts for *RET*-mutant MTC, *RET*-fusion non-small-cell lung cancer, and other RET-altered malignancies, including those previously treated with selective RET inhibitors. By sparing VEGFR2 and offering favorable pharmacokinetic and pharmacodynamic profiles, BOS172738 aims to achieve full RET inhibition with a more tolerable safety profile, supporting its potential as a next-generation targeted therapy.^32^ At the 2021 ASCO meeting, early efficacy results were presented: among 67 patients with RET-altered tumors, BOS172738 demonstrated an ORR of 44% in *RET*-mutant MTC (including one complete response). Treatment was generally well tolerated, with most AEs grade ≤2, most commonly creatine phosphokinase elevation, fatigue, constipation, and anemia. Median DOR had not yet been reached at the data cutoff (longest ongoing response 659 days), supporting durable activity.^33^

#### TAS0953/HM06

The MARGARET study (NCT04683250) is an ongoing phase I/II clinical trial evaluating the use of the selective RET inhibitor TAS0953/HM06 in adults (18 years and older) with advanced or metastatic solid tumors harboring RET mutations or fusions, regardless of prior MKI treatment. The initial phase focuses on identifying the optimal dosage and establishing safety thresholds, while the second phase centers on assessing the drug’s efficacy, with ORR as the primary endpoint.^34^

#### SY-5007

SY-5007 is a novel, selective RET inhibitor designed to overcome resistance mutations such as *RET* M918T and solvent front mutations (SFMs). In a first-in-human phase 1 trial,^35^ 23 patients with *RET*-mutant MTC were treated with SY-5007. At the recommended phase 2 dose of 160 mg BID, the ORR was 52.2% and the DCR was 91.3%. Among treatment-naïve MTC patients, the ORR reached 75.0% (vs 40.0% in previously treated patients). Median PFS and DOR were not reached at the median follow-up of 6.44 months, and the estimated 12-month PFS was 85.2%. These results suggest SY-5007 is a promising RET-targeted therapy in MTC, warranting further investigation.

#### ^177^Lu-DOTATATE peptide receptor radionuclide therapy (PRRT)

Several studies have explored the role of ^177^Lu-DOTATATE PRRT in MTC. Parghane et al.^36^ evaluated 43 patients with somatostatin receptor–positive (SSTR-positive) MTC treated with ^177^Lu-DOTATATE PRRT. They reported median PFS of 24 months and OS of 26 months. Patients who had calcitonin doubling time (CtnDT) > 24 months showed longer OS (median PFS not yet reached vs 10 months and median OS 60 months vs 20 months). PRRT was well tolerated, with no major grade 3-4 toxicity.

Similarly, Satapathy et al.^37^ studied 8 advanced MTC patients receiving ^177^Lu-DOTATATE combined with low-dose capecitabine. SD was seen in 86%, with molecular response, as per the European Organization for Research and Treatment of Cancer criteria, in all. Biochemical response with reduction in serum Ctn levels was observed in 60%. Toxicity was minimal. ^177^Lu-DOTATATE PRRT is a promising and well-tolerated treatment option in SSTR-positive MTC, warranting further prospective evaluation.

#### CAR-T cell therapy

A phase I clinical trial (NCT04877613) is investigating the safety and feasibility of GFRα4-directed CAR-T cell therapy in patients with recurrent or metastatic MTC who have progressed on or declined prior TKI therapy. This open-label, dose-escalation study utilizes autologous T-cells genetically engineered to express a single-chain variable fragment targeting GFRα4, in combination with a 4-1BB/CD3ζ co-stimulatory domain. Patients receive a single infusion following lymphodepletion with fludarabine and cyclophosphamide. The study’s primary objective is to determine the maximum tolerated dose, while secondary outcomes include response rates, DOR, PFS, and OS.^38^

#### Clinical biomarkers

Recent advances have explored circulating cell-free DNA (cfDNA) as a potential biomarker in MTC. Citarella et al. investigated cfDNA fragmentation and methylation patterns using droplet digital PCR in plasma samples from MTC patients. They found that the short fragment fraction was significantly elevated in patients with active disease compared to healthy controls, with an area under the receiver operating characteristic curve of 0.87, suggesting good discriminatory ability. In parallel, hypermethylation of a CpG site in the MGMT gene (*CG_16698623*) was observed in MTC patients, including those with low or borderline Ctn levels, achieving an AUC of 0.89. Importantly, neither marker correlated with serum Ctn, highlighting their potential utility in poor Ctn-producing tumors. These findings support the role of cfDNA-based assays as emerging noninvasive tools for disease detection and monitoring.^39^ Emerging data using personalized tumor-informed ctDNA assays, Signatera, have demonstrated feasibility in thyroid cancers, with high specificity and positive predictive value for disease detection and surveillance, although current evidence in MTC remains limited by small sample sizes and preliminary reports.^40^

Personalizing MTC therapy may also involve patient-related biomarkers. Dalmiglio et al.^41^ assessed the “Controlling Nutritional Status (CONUT) score” in 42 advanced thyroid cancer patients (11.9% MTC) treated with TKIs. The CONUT score is a nutritional screening tool based on 3 components: serum albumin, total cholesterol, and total lymphocyte count. Each component is scored separately: serum albumin (0-6 points), total cholesterol (0-3 points), and total lymphocyte count (0-3 points), and then summed to generate a total score (0-12). A score of 0 indicates normal nutritional and immune status, while higher scores reflect increasing degrees of malnutrition and immunosuppression. Patients were stratified into 3 groups according to CONUT score: Group A (CONUT score 0-2), Group B (CONUT score 3-4), and Group C (CONUT score 5-7). PFS and overall survival was significantly better in group A. Thus, an elevated CONUT could predict worse outcomes on TKIs, suggesting that nutritional status could help risk-stratify patients before therapy. Incorporating such screening tools into trials with larger sample sizes could refine patient selection or indicate the need for supportive interventions.

#### Resistance

Gatekeeper mutations represent an important resistance mechanism in MTC, particularly at RET V804L and V804M. These mutations introduce bulkier side chains at the ATP-binding cleft, which hinder the binding of MKIs like vandetanib and cabozantinib. While selective RET inhibitors such as selpercatinib and pralsetinib were designed to bypass V804-mediated steric hindrance, co-occurrence of gatekeeper and solvent front mutations (SFMs) can still compromise efficacy.^20^

SFMs occurring within the RET kinase domain pose a major challenge by reducing the effectiveness of targeted therapies in MTC. These alterations can disrupt drug binding and facilitate development of resistance.^20^^,^^42^ SFMs occur at residues positioned near the solvent-exposed regions of the kinase, altering the binding affinity of kinase inhibitors and conferring resistance. Common SFMs identified in MTC patients include mutations at residue *RET* G810, such as G810C/S, as well as alterations at the *RET* Y806 hinge region (Y806C/N).^42^ These mutations have been documented following treatment with highly selective RET inhibitors, such as selpercatinib and pralsetinib.^20^^,^^42^

Clinical observations highlight that both MKIs, cabozantinib and vandetanib, and selective RET inhibitors, selpercatinib and pralsetinib, exhibit significantly reduced efficacy against SFMs.^20^^,^^42^^,^^43^ Specifically, in clinical cases, patients initially responding to selpercatinib developed resistance with detectable SFMs, manifesting clinically as disease progression. Preclinical studies reinforce these findings, confirming that cells harboring SFMs like *RET* G810C exhibit drastically increased IC50 values against selpercatinib compared to wild-type *RET*, thus validating the clinical resistance phenomenon.^42^

Several next-generation RET inhibitors, such as TPX-0046 and BOS172738, are in development to overcome resistance. TPX-0046 shows preclinical potency against SFMs but lacks efficacy against gatekeeper mutations, while BOS172738 demonstrates inhibitory activity against both SFMs and V804L/M gatekeeper variants.^20^ Additionally, combinatorial treatment strategies may be further investigated to counteract resistance mechanisms bypassing RET signaling altogether.^43^ Ongoing research is critical to enhance the therapeutic arsenal against *RET*-driven MTC.

### From trials to treatment: practical implications for MTC

Many patients with metastatic MTC have indolent disease and can be safely observed before initiating systemic therapy. Localized approaches, including surgery, external beam radiation, ablation, or embolization are preferred for an isolated, progressive lesion that is symptomatic or at risk of compromising/invading a nearby vital structure. For instance, a painful vertebral metastasis at risk of causing cord compression, or a neck mass threatening the airway. Before starting systemic treatment, molecular status guides drug choice. For *RET*-mutant disease, selpercatinib is now the preferred first-line therapy, offering higher response rates and longer PFS than MKIs. If disease subsequently progresses, repeat biopsy of growing target lesion or liquid biopsy may identify resistant mutations. Therapy is then typically switched to a MKI such as vandetanib or cabozantinib. In *RET*-wild type disease, vandetanib and cabozantinib remain standard first-line options, with the choice shaped by comorbidities and toxicity profiles: vandetanib requiring ECG monitoring for QTc prolongation and cabozantinib more often limited by gastrointestinal side effects and weight loss. Vandetanib is contraindicated in patients with baseline QTc prolongation and carefully used in patients having electrolyte abnormalities (hypokalemia, hypomagnesemia) from persistent MTC induced diarrhea. It is preferable not to use cabozantinib in patients at risk of fistula formation (for instance, history of Crohn’s disease, or advanced neck disease with previous localized radiotherapy).

A special circumstance is when there is *RET* positive advanced, progressive, unresectable locoregional neck disease, where selpercatinib becomes an option in the neoadjuvant setting—with or without distant metastasis.^44^

The goal is to delay the need to start systemic therapy as much as possible for the following reasons: (1) treatment is lifelong (interrupted only if medication is not tolerated due to AEs, or if the drug stops being effective as shown by disease progression on re-staging), (2) risk of AEs (especially when patients are asymptomatic prior to starting systemic therapy compromising the quality of life), (3) development of new resistance mechanisms rendering the drug ineffective with subsequent early exhaustion of lines of treatment options, and/or (4) more frequent clinic visits with the respective increase in socio-economic burden to patient and family.

In practice, therefore, localized therapy should be pursued where feasible, while systemic therapy is reserved for advanced, oligometastatic disease that is unresectable, progressive, and/or symptomatic; or in the neoadjuvant setting for locoregionally advanced RET-mutated unresectable disease. The *RET* status determines whether a selective RET inhibitor or MKI is most appropriate as first-line option.^45^

#### Summary

MTC is a rare thyroid cancer that historically has limited therapeutic options due to its resistance to conventional chemotherapy and radioactive iodine being ineffective. Recently, tyrosine kinase targeted therapies have significantly advanced treatment outcomes. Initially, the MKIs vandetanib and cabozantinib improved disease control but came with notable side effects and development of tumor resistance mechanisms leading to disease progression. More recently, selective RET inhibitors have shown higher efficacy and tolerability, becoming preferred options in RET-mutant MTC despite pralsetinib’s recent market withdrawal due to regulatory issues. Emerging therapies, including newer selective RET inhibitors and PRRT, show promising early results. Continued research is essential for addressing resistance and further improving patient outcomes in advanced MTC.

## Data Availability

This is a narrative review based on previously published studies. No new datasets were generated or analyzed for this manuscript. All data supporting the findings of this study are available within the cited references.
